# Exploring and comparing renal adverse effects between PARP inhibitors based on a real-world analysis of post-marketing surveillance data

**DOI:** 10.3389/fmed.2024.1412700

**Published:** 2024-10-18

**Authors:** Qiuyu Xu, Lin Jiang, Gang Chen, Sanxi Ai, Xiaohong Fan, Gangan Wang, Chunyu Jia, Jiahui Wang, Ke Zheng, Bin Zhao, Yan Qin, Xuemei Li

**Affiliations:** ^1^Department of Nephrology, Peking Union Medical College Hospital, Peking Union Medical College, Chinese Academy of Medical Sciences, Beijing, China; ^2^Department of Pharmacy, Peking Union Medical College Hospital, Peking Union Medical College, Chinese Academy of Medical Sciences, Beijing, China

**Keywords:** onco-nephrology, nephrotoxicity, renal function, ovarian cancer, adverse event reporting system

## Abstract

**Objective:**

Poly (ADP-ribose) polymerase inhibitors (PARPis) are emerging targeted therapeutic agents in oncology, primarily indicated for ovarian and metastatic breast cancer. Acute kidney injury (AKI) has been observed in patients undergoing PARPi treatment, while there is still a lack of comprehensive comparisons of AKI associated with different PARPis. Our study aimed to extensively characterize the renal adverse effects (RAEs) of PARPi using real-world data.

**Methods:**

Disproportionality analysis and Bayesian analysis were employed for data mining to identify suspected RAE cases after different PARPis use within the Food and Drug Administration’s Adverse Event Reporting System from January 2004 to September 2023. The time to onset, fatality, and hospitalization rates of PARPi-related RAEs were also investigated.

**Results:**

We identified 1,696 PARPi-related RAEs, predominantly affecting patients over 85 (56.31%). Veliparib exhibited a more pronounced association with RAEs compared to others, as indicated by the highest reporting odds ratio (ROR = 29.20, 95% CI = 8.79–96.97), proportional reporting ratio (PRR = 19.80, χ^2^ = 72.62), and empirical Bayes geometric mean (EBGM = 19.80, the lower 90% one-sided CI = 7.25). The median time to RAEs onset was 15 (interquartile range: 6–55.75) days following the initiation of PARPi therapy. PARPi-related RAEs generally led to a 28.15% hospitalization rate and a 4.34% fatality rate.

**Conclusion:**

Although the majority present with reversible creatinine elevation, PARPi-related RAEs merits broader attention, given its potential for clinical consequences. We should strive to early identify those individuals who may have irreversible kidney damage. The focus should be directed toward monitoring renal function in individuals receiving PARPi, especially in senile people and those with a predisposition to AKI.

## 1 Introduction

Poly ADP-ribose polymerase (PARP) is crucial in DNA repair after a damaging event (1). As the first targeted drugs influencing DNA damage response, PARP inhibitors (PARPis) have instigated a transformative era in oncology (2), particularly within the realm of ovarian cancer. Their mechanism is to selectively eliminate cells with homologous recombination deficiency by operating on synthetic sickness, which occurs when the deficiency of two or more cellular mechanisms results in cell death (3, 4). In 2014, Olaparib (5), as the first drug of PARPi, received approval for maintenance therapy in platinum-sensitive advanced ovarian cancer with germline mutations in DNA repair genes *BRCA 1/2*. Subsequently, the Food and Drug Administration (FDA) has approved many PARP inhibitors, including Olaparib, Niraparib (6), Rucaparib (7), and Talazoparib (8), with indications spanning ovarian, prostate, pancreatic, breast, and other cancer types (9). Moreover, besides the four PARPis mentioned above, several other PARPis such as Pamiparib (10) and Fluzoparib (11), have obtained approval in countries outside the United States. According to the latest National Comprehensive Cancer Network guidelines, PARP inhibitors are recommended as the first-line choice for maintenance therapy following initial chemotherapy in ovarian cancer regardless of *BRCA* genotype (12, 13).

The widespread use of PARP inhibitors has prompted increased scrutiny of drug-related adverse events (AEs). In pivotal PARPi clinical trials, hematologic toxicities were the most common AEs that have led to dose modifications. Other commonly observed AEs encompass gastrointestinal disorders, photosensitivity, elevated creatinine, and fatigue (14). Notably, in the clinical trial involving Olaparib, the proportion of increased creatinine levels in the Olaparib group is 11% versus 1% in the placebo group (15). In the context of cancer patients, acute kidney injury (AKI) warrants particular attention, with a crucial focus on AKI related to targeted treatments (16, 17). Reduction in kidney function not only can impede the timely and effective implementation of appropriate cancer therapies but is also associated with a notable increase in hospitalization and mortality rates (18, 19). There is a growing number of research and post-marketing pharmacovigilance data on PARPi-associated AKI during these years (20–23), along with documented case reports where substantive evidence of renal pathological damage exists (24).

Although some studies suggest that the increase in creatinine after PARPi administration is relatively mild and reversible (23, 25), there is currently a lack of large-scale cohort studies and real-world data confirming this perspective. The potential for drug-related AKI remains a reason for some patients to discontinue the medication. Moreover, some patients experience difficulties partially or fully restoring creatinine to baseline after discontinuation (22, 23, 26). These aspects indicated a limited understanding and systematic exploration of large-scale renal adverse effects (RAEs) in the real world. Our study aimed to evaluate and compare the associations between different PARP inhibitors and RAEs in a large population by investigating the FDA’s Adverse Event Reporting System (FAERS). Since the inherent limitations in the FAERS database prevent access to baseline renal function information and hinder our ability to ascertain whether the reports strictly align with The Kidney Disease: Improving Global Outcomes (KDIGO)-defined AKI criteria (27), we referred to those events reported as “PARPi-related RAEs” in the following discussion. Additionally, our investigation delved into the onset time, fatality rates, and hospitalization rates for RAEs across different PARPi regimens.

## 2 Materials and methods

### 2.1 Data source

The retrospective pharmacovigilance study utilized data from the FAERS database from January 2004 to September 2023. As a global spontaneous reporting system (SRS), FAERS collects drug AE reports from healthcare professionals, patients, and pharmaceutical manufacturers globally (28, 29). The FAERS data files encompass a comprehensive array of information, including demographic and administrative details (DEMO), drug information (DRUG), preferred terms (PTs) coded for the adverse events (REAC), patient outcomes (OUTC), report sources (RPSR), therapy start dates and end dates for reported drugs (THER), and indications for drug administration (INDI).

**FIGURE 1:**
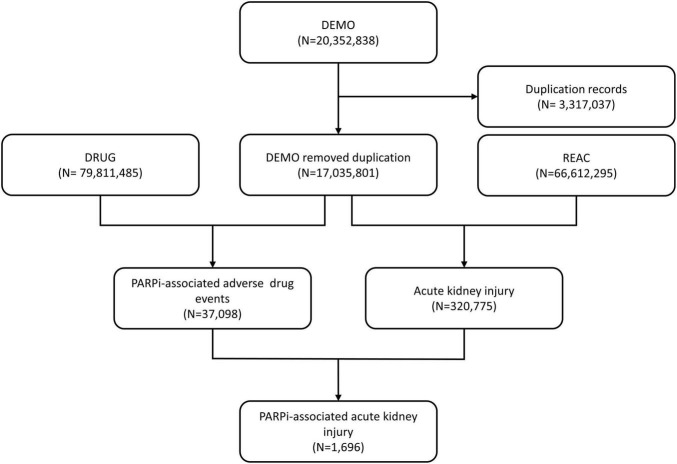
Process of the selection of cases of PARPi-associated acute kidney injury from the FAERS database.

Within the FAERS database, AEs are coded based on event-related information according to the Medical Dictionary for Regulatory Activities (MedDRA), which is a hierarchical dictionary serving as a coding system for diagnoses, symptoms and signs, investigations, surgical and medical procedures, therapeutic indications, and medical history (30). An initial screening involved analyzing 20,352,838 AE reports retrieved from the FAERS database. Following FDA guidance, we identified and discarded redundancy by selecting the latest FDA_DT in cases where CASEID and FDA_DT matched. Subsequently, our dataset for further analysis comprised 17,035,801 reports (Figure 1).

### 2.2 AE and drug identification

Utilizing MedDRA (version 26.0) at the PT level, we conducted a comprehensive search within the REAC files to identify terms associated with RAEs. The following PTs were carefully considered in the context of RAEs, mainly when administered with PARP inhibitors: “acute kidney injury,” “blood creatinine increased,” “blood urea abnormal,” “glomerular filtration rate decreased,” “renal impairment,” “oliguria,” “anuria,” “dialysis,” “hemodialysis,” “peritoneal dialysis,” “nephropathy toxic,” and “tubulointerstitial nephritis.” Furthermore, we meticulously selected the generic and brand names of PARP inhibitors using http://www.drugbank.ca as the reference dictionary in the data mining process (Table 1).

**TABLE 1:** Summary of poly ADP-ribose polymerase (PARP) inhibitors.

Generic name	Brand name/development code	Year of approval by FDA
Olaparib	Lynparza	2014
Niraparib	Zejula	2017
Rucaparib	Rubraca	2016
Talazoparib	Talzenna	2018
Veliparib	ABT-888	Not yet
Pamiparib	BaiHuiZe/BGB-290	Not yet
Fluzoparib	AiRuiYi/SHR-3162	Not yet

### 2.3 Data mining

Data mining algorithms have been developed to detect signals of drug-associated AEs, identifying patterns reported more frequently than anticipated by estimating expected reporting frequencies based on information on all drugs and all events in the database (31, 32). To examine the potential association between the administered drug and the occurrence of specific AEs, our analysis incorporated multiple algorithms based on the rationale of Bayesian analysis and disproportionality analysis. Four distinct algorithms including reporting odds ratio (ROR) (33), proportional reporting ratio (PRR) (34), Bayesian confidence propagation neural network (BCPNN) (35), and multi-item gamma Poisson shrinker (MGPS) (36) are widely used (Table 2). They are employed by the Netherlands Pharmacovigilance Centre, the Medicines and Healthcare Products Regulatory Agency of the United Kingdom, the World Health Organization, and the FDA, respectively.

**TABLE 2:** Summary of major algorithms used for signal detection.

Algorithms	Equation*	Criteria
ROR	ROR = (a / b) / (c / d)	95% CI > 1, *N* ≥ 2
	95% CI = e^ln(ROR) ± 1.96(1 / a + 1 / b + 1 / c + 1 / d)∧0.5^	
PRR	PRR = (a / (a + c)) / (b / (b + d))	PRR ≥ 2, χ^2^ ≥ 4, *N* ≥ 3
	χ^2^ = Σ((O − E)2 / E); (O = a, E = (a + b)(a + c) / (a + b + c + d))	
BCPNN	IC = log_2_a(a + b + c + d) / ((a + c)(a + b))	IC025 > 0
	IC025 = e^ln(IC)–1.96(1 / a + 1 / b + 1 / c + 1 / d)∧0.5^	
MGPS	EBGM = a(a + b + c + d) / ((a + c)(a + b))	EBGM05 > 2, *N* > 0
	EBGM05 = e^ln(EBGM)–1.64(1 / a + 1 / b + 1 / c + 1 / d)∧0.5^	

ROR, reporting odds ratio; CI, confidence interval; *N*, the number of co-occurrences; PRR, proportional reporting ratio; χ^2^, chi-squared; BCPNN, Bayesian confidence propagation neural network; IC, information component; IC025, the lower limit of the 95% two-sided CI of the IC; MGPS, multi-item gamma Poisson shrinker; EBGM, empirical Bayesian geometric mean; EBGM05, the lower 90% one-sided CI of EBGM.

*a, number of reports containing both the suspect drug and the suspect adverse drug reaction; b, number of reports containing the suspect adverse drug reaction with other medications (except the drug of interest); c, number of reports containing the suspect drug with other adverse drug reactions (except the event of interest); d, number of reports containing other medications and other adverse drug reactions.

Our inquiry focused on a comparative analysis of the associations between RAEs and different PARP inhibitors, identified as “PS” (primary suspect) in the ROLE_COD field of DRUG files. We also estimated the onset time of RAEs for various PARP inhibitors. This was defined as the duration between the EVENT_DT (adverse event onset date) and the START_DT (initiation date of administration). Records with incorrect entries or erroneous input, where EVENT_DT preceded START_DT, were excluded from the analysis. Additionally, we evaluated reports involving fatal events attributed to adverse drug reactions. The fatality rate was calculated by dividing catastrophic events by the total number of RAEs associated with PARP inhibitors.

### 2.4 Statistical analysis

Descriptive analysis was employed to summarize the clinical characteristics of RAE patients due to PARP inhibitors from the FAERS database. For evaluating differences in the onset times of RAEs among various PARP inhibitors, we employed nonparametric tests (the Mann–Whitney test for dichotomous variables and the Kruskal–Wallis test when there were more than two subgroups of respondents). Hospitalization and fatality rates between distinct PARP inhibitors were compared using Pearson’s Chi-squared test or Yates’ continuity correction Chi-squared test (when expected frequencies were less than five). Statistical significance was established at *P* < 0.05, maintaining 95% confidence intervals. Data mining and statistical analyses were performed using SAS, version 9.4 (SAS Institute Inc.).

## 3 Results

### 3.1 Descriptive analysis

About 37,098 AEs linked to PARP inhibitors and 320,775 reports associated with RAEs were documented in the FAERS database, dated from January 2004 to September 2023 (Figure 1). Of these, we have screened the intersection of the two sets, identified 1,696 reports with suspected PARPi-related AEs, and summarized the clinical features of these patients in Table 3. Most cases were reported from North America (71.70%), followed by 19.16% from Asia. The reported cases of RAEs have gradually increased from 2015 to 2019 and remained at nearly 300 cases per year since then. Although most patients eligible for PARP inhibitors were females, 2.89% of the reports were from male patients. Notably, a significant portion of the reports originated from the very elderly population, with individuals over 85 years old accounting for 56.31% of the cases. The age distribution among the remaining was relatively balanced, with 16.27% of the reports from those aged 45–64, 17.63% from those aged 65–74, and 8.84% from those aged 75–84. Niraparib generated the most significant number of RAE reports (*N* = 1,034, 60.97%) in our study, followed by Rucaparib (*N* = 365, 21.52%) and Olaparib (*N* = 282, 16.63%). Regarding the indications for PARPi leading to RAEs, PARP inhibitors were primarily prescribed in patients with ovarian cancer (*N* = 1,311, 79.89%), and prostate cancer-related cases accounted for 2.44% of the total. For clinical outcomes, the rates of initialed or prolonged hospitalization and mortality stood at 28.15% and 4.34%, respectively.

**TABLE 3:** Clinical characteristics of patients with PARPi-related AKI sourced from the FAERS database (January 2004 to September 2023).

Characteristics	Reports, no. (%)
**Reporting region**	
North America	1,216 (71.70)
Europe	137 (8.08)
Asia	325 (19.16)
South America	4 (0.24)
Africa	1 (0.06)
**Reporting year**	
2015	16 (0.94)
2016	17 (1.00)
2017	96 (5.66)
2018	290 (17.10)
2019	241 (14.21)
2020	214 (12.62)
2021	290 (17.10)
2022	299 (17.63)
2023 (Q1–Q3)	233 (13.74)
**Sex of patients**	
Male	49 (2.89)
Female	1,152 (67.92)
Unknown or missing	495 (29.19)
**Age groups (years)**	
30–44	16 (0.94)
45–64	276 (16.27)
65–74	299 (17.63)
75–84	150 (8.84)
>85	955 (56.31)
**PARPi as suspected drug**	
Olaparib	282 (16.63)
Niraparib	1,034 (60.97)
Rucaparib	365 (21.52)
Talazoparib	11 (0.65)
Veliparib	4 (0.24)
**Indications**	
Ovarian cancer	1,311 (79.89)
Fallopian tube carcinoma	76 (4.63)
Uterine carcinoma	13 (0.79)
Cervix carcinoma	4 (0.24)
Other gynecological malignant neoplasm	4 (0.24)
Breast cancer	16 (0.98)
Prostate cancer	40 (2.44)
Lung cancer	3 (0.18)
Digestive system malignant neoplasm	11 (0.67)
Peritoneal malignant neoplasm	43 (2.62)
Bladder cancer	1 (0.06)
Neoplasm with unclear primary sites	119 (7.25)
**Outcomes**	
Congenital anomaly	1 (0.07)
Hospitalization	402 (28.15)
Life-threatening	71 (4.97)
Disability	12 (0.84)
Death	62 (4.34)
Other serious/important medical event	1,296 (90.76)

PARPi, poly ADP-ribose polymerase inhibitor; AKI, acute kidney injury; FAERS, Food and Drug Administration’s Adverse Event Reporting System.

### 3.2 Disproportionality analysis and Bayesian analysis

We detected RAE signals for five PARP inhibitors based on the criteria for the four algorithms (Table 2) and listed the results in Table 4. AKI in all PARP inhibitors except Talazoparib was overreported compared to the background frequency. Although it contributed the lowest percentage of reports, Veliparib was noteworthy for its relationship to AKI amongst various PARP inhibitors due to its highest ROR (ROR = 29.20, 95% CI = 8.79–96.97), PRR (PRR = 19.80, χ^2^ = 72.62), and empirical Bayes geometric mean (EBGM = 19.80, the lower 90% one-sided CI = 7.25). Among FDA-approved PARP inhibitors, Niraparib ranked the first, with ROR of 3.63 (95% CI = 3.41–3.87), PRR of 3.48 (χ^2^ = 1,851.32), and EBGM of 3.47 (the lower 90% one-sided CI = 3.29). In contrast, Olaparib, the first approved PARPi, exhibited a weaker association with RAEs, suggesting its relative renal safety profile.

**TABLE 4:** Association of different PARP inhibitors regimens with AKI.

Drug	*N*	ROR (95% two-sided CI)	PRR (χ^2^)	IC (IC025)	EBGM (EBGM05)
Olaparib	292	1.58 (1.40, 1.78)	1.57 (58.52)	0.65 (0.57)	1.56 (1.42)
Niraparib	1,203	3.63 (3.41, 3.87)	3.48 (1,851.32)	1.80 (1.69)	3.47 (3.29)
Rucaparib	324	2.83 (2.55, 3.14)	2.75 (411.70)	1.46 (1.31)	2.74 (2.51)
Talazoparib	21	0.83 (0.46, 1.51)	0.84 (0.36)	−0.26	0.84 (0.51)
Veliparib	4	29.20 (8.79, 96.97)	19.80 (72.62)	4.31 (1.30)	19.80 (7.25)

PARP, poly ADP-ribose polymerase; AKI, acute kidney injury; *N*, the number of reports of PARPi-related AKI; ROR, reporting odds ratio; CI, confidence interval; PRR, proportional reporting ratio; χ^2^, chi-squared; IC, information component; EBGM, empirical Bayes geometric mean.

### 3.3 Time to onset of PARPi-associated renal adverse effects

**FIGURE 2:**
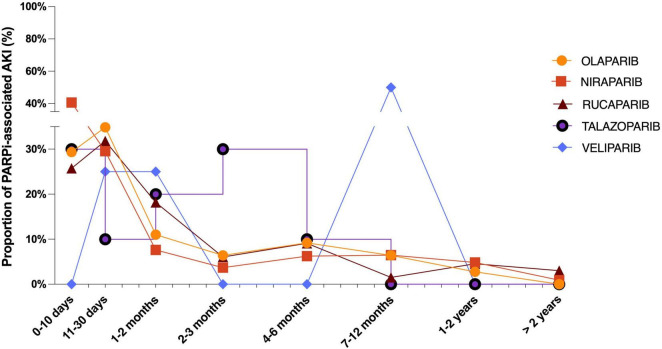
Time to event onset of acute kidney injury following PARP inhibitor regimens.

The median time to onset of PARPi-associated RAEs was 15 days [interquartile range (IQR): 6–55.75 days] in general. The time to RAE onset for each PARPi is depicted in Figure 2. We observed that nearly 90% of RAE cases could occur within the first 3 months after PARPi administrations. RAEs could occur as soon as the first dose after Olaparib, Niraparib, Rucaparib, and Talazoparib, indicated by the identical dates of drug administration and RAE onset in the database. We identified a significant difference in time to RAEs among all PARPi regimens (Kruskal–Wallis test, *P* = 0.027). The median times to RAE onset among different PARP inhibitors were 18 (IQR: 7–57) days for Olaparib, 14 (IQR: 5–67.45) days for Niraparib, 24 (IQR: 10.25–58.75) days for Rucaparib, 39 (IQR: 6.75–77.25) days for Talazoparib, and 179 (IQR: 41.75–312) days for Veliparib, respectively.

### 3.4 Fatality and hospitalization due to PARPi-associated renal adverse effects

**FIGURE 3:**
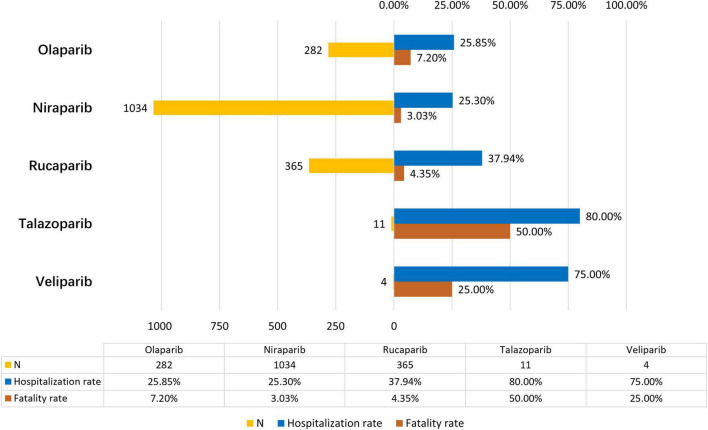
The number of reports, hospitalization rates, and fatality rates for PARPi-associated acute kidney injury.

To evaluate the prognosis of PARPi-associated RAEs, we assessed the fatality and hospitalization rates resulting from RAEs associated with different PARPi treatments and generated Figure 3. In our analysis, we found the outcome of PARPi-associated RAEs tended to be poor, generally resulting in 28.15% hospitalization and 4.34% death. Significant variation in hospitalization rates was observed across different PARPi regimens as determined by Pearson’s chi-square test for overall comparison (*P* < 0.001). As for fatality rates, considering the limited number of deaths associated with Talazoparib and Veliparib-related RAEs, we utilized Yates’ continuity correction Chi-squared test to compare the difference in fatality rates among all five regimens, and the results were statistically significant (*P* < 0.001). Specifically, Talazoparib exhibited the highest hospitalization rate (80%) and mortality rate (50%) despite having the lowest ROR. Additionally, for the three PARP inhibitors (Olaparib, Niraparib, and Rucaparib) widely used in real-world settings with more extended usage experience, the fatality rates of their associated RAEs still showed significant differences (assessed by Pearson’s Chi-square test, *P* < 0.001). The lowest hospitalization (25.3%) and fatality rates (3.03%) were observed in cases linked to Niraparib, although it has relatively high ROR, PRR, and EBGM.

## 4 Discussion

To our knowledge, this study is the first and most extensive collection until recently to compare the associations, timing, and prognosis of RAEs after different PARP inhibitors in real-world practice based on the FAERS pharmacovigilance database. Our findings align with conclusions drawn in previous research, confirming that PARP inhibitors can indeed trigger RAEs, emphasizing the significance of this phenomenon. The extended temporal coverage, a large number of reported cases globally, and lack of restrictions to specific timeframes and conditions, as seen in randomized controlled trials (RCTs), distinguish our study as a necessary supplement to prior research.

Our study observed a robust correlation between PARPi and RAEs, with diverse strengths among different PARPi regimens. Among widely used PARP inhibitor regimens, Olaparib exhibited the weakest association with RAEs and relatively lower hospitalization and mortality rates, indicating its comparatively higher renal safety. Specifically, we observed that RAEs related to Olaparib were less common in individuals aged 85 and older (34.75%) compared to the overall PARPi-associated RAEs reports (56.31%). However, due to the absence of comprehensive data on the age distribution of the total patient population using each PARPi, we cannot ascertain whether the lower AKI risk associated with Olaparib is a result of its less frequent use in older populations. Veliparib demonstrated stronger RAE signals despite the relatively limited number of reports. In RCTs targeting the primary indications of PARPi, reports of adverse reactions related to AKI are notably common, with variations observed among different regimens. In the SOLO2 trial, 11% of patients treated with Olaparib reported elevation in creatinine compared with 1% in the placebo group (15). Similarly, the NOVA trial indicated that 11% of patients treated with Niraparib experienced the increase of blood creatinine, while this ratio was 4% for the placebo group (37). Rucaparib use in the ARIEL3 trial resulted in an elevation of creatinine in 15% of patients versus 2% in the placebo group (7). In our current study, more than half of AKI reports came from the very elderly population, with those over 85 years old providing 56.31% of the reports. Advanced age is usually associated with poorer baseline renal function and underlying renal diseases (38). This underscores that in populations with pre-existing renal impairment or using other potentially nephrotoxic drugs, PARP-related RAEs are more likely to occur, aligning with inferences from previous research (23, 24).

Currently, there remains some controversy surrounding PARPi-related creatinine elevation, with some studies proposing that PARP inhibitors may elevate creatinine levels by affecting the renal transporters that constitute the tubular creatinine secretion pathway (39) rather than causing a genuine decline in glomerular filtration rate (40). In our investigation, reports submitted for adverse reactions primarily attributed the occurrence of RAEs to PARP inhibitors, as we selected those reports in which PARPi was the Primary Suspect drug. Notably, more than half of the reports originated from the very elderly population, with those over 85 accounting for 56.31%. This demographic is often underrepresented in clinical trials, making it especially important to take special care when considering this population. These individuals usually face more vulnerable physiological conditions and complex comorbidities, leading to more significant challenges in treatment decisions. Furthermore, this age group is more susceptible to worsening creatinine levels or AKI due to various factors (41). These factors often include common side effects of PARPi (14), such as nausea, vomiting, anorexia, diarrhea, or interactions with other medications. This susceptibility likely explains the relatively severe clinical outcomes observed in our study than former reported clinical trials, including higher rates of hospitalization and fatality associated with PARPi-related RAEs.

Our findings suggest that while PARPi-related RAEs primarily manifest as reversible increases in creatinine, they may not be entirely reversible in certain populations, particularly among the elderly and those more vulnerable to renal injury. PARPi-related RAEs can lead to significant clinical consequences, including increased hospitalization rates and, to some extent, higher mortality. This increased risk is likely influenced by multiple factors, such as advanced age and comorbidities, rather than directly attributable to PARPi-related RAEs alone. In our collected reports, we identified cases diagnosed as tubulointerstitial nephritis, which posed significant feasibility and risk-benefit concerns in patients with advanced cancer. It is possible that a proportion of the patients with elevated creatinine levels may also have underlying substantive renal damage. Therefore, these RAE events should not be regarded as entirely benign and should not be overlooked. Considering both our data and previous study indicate that PARPi-related RAEs frequently occur within the first 30 days of treatment, proactive renal function monitoring during this critical period is essential, particularly for high-risk populations (23). In addition to serum creatinine, attention should also be directed toward cystatin C, markers of tubulointerstitial injury, and other novel emerging biomarkers, such as kidney injury molecule-1 (KIM-1), neutrophil gelatinase-associated lipocalin (NGAL), N-acetyl-β-D-glucosaminidase (NAG), and tissue inhibitor of metalloproteinase-2 (TIMP-2) times insulin-like growth factor-binding protein 7 (IGFBP7) (42, 43).

The sensitivity of recurrent ovarian cancer to adjunctive treatments beyond chemotherapy remains an essential factor associated with the progression-free survival of ovarian cancer patients (44, 45). Restrictions on medication use post-AKI can detrimentally affect tumor prognosis (14). Additionally, many drugs undergo renal metabolism, and declining renal function necessitates adjustments not only in cancer therapies but also in various other medications, especially in the elderly population (18, 46). Given the complexities of advanced cancer treatment and the scarcity of renal biopsy in such patients, a comprehensive understanding of the mechanisms of action, indications, and side effects of PARP inhibitors is crucial. On the other hand, timely adjustment of PARPi dosage based on renal function is important. According to the latest guidelines and United States Prescribing Information, for patients with moderate renal impairment (creatinine clearance: 30–59 ml/min), dose adjustment is not required for Niraparib and Rucaparib, whereas the dose of Olaparib must be adjusted according to renal function at the start of treatment (13, 47). In cases of severe renal impairment (creatinine clearance < 30 ml/min), prospective studies and case reports suggest dose adjustment strategies for Olaparib and Niraparib (48, 49). However, there is a lack of clinical data for Rucaparib in this population, and its use should be limited to situations where the potential benefits clearly outweigh the risks.

Despite leveraging real-world data in this study, our study also has limitations to consider. Firstly, analyzing adverse drug reaction signals within the SRS has inherent constraints. Due to the voluntary nature of the reporting system, the SRS database is susceptible to underreporting and selection bias. The methodology estimates the comparative reporting rates for specific reactions to individual drugs, which may be influenced by external factors, and reporting tendencies can vary among different populations (31, 50). Secondly, we also observed imperfections in the data, such as incorrect inputs and incomplete reports, which could introduce bias into the analysis. Furthermore, profiling specific and critical risk factors between PARP inhibitors and RAEs is challenging due to the lack of baseline renal function data, pre-existing renal diseases, previous and current chemotherapy, and other comorbidities that might influence RAEs. These constraints also impede our ability to accurately assess the degree of AKI according to KDIGO guidelines. Consequently, the causality between PARPi-related RAEs and their clinical outcomes may be influenced by multiple unknown factors. Therefore, the data mining results should not be interpreted as definitive evidence of a causal relationship but rather as signals that warrant further investigation through well-organized clinical studies.

## 5 Conclusion

Based on our pharmacovigilance analysis in the FAERS database, RAE is a common side effect of PARP inhibitors. The mechanisms underlying the increase in serum creatinine levels induced by PARPis remain unclear, and different PARPis may involve partially distinct mechanisms, as evidenced by our results showing variations in the incidence of PARPi-associated RAEs. Hence, we propose that the elevation of creatinine levels following PARPi administration should not be simplistically defined as pseudo-AKI. Such categorization may obscure potential renal injury and lead to an irreversible decline in kidney function. Therefore, a comprehensive assessment is crucial for patients experiencing creatinine level elevation to differentiate AKI. Identifying relevant factors contributing to RAEs enables better discrimination of cases requiring timely intervention. Whereas in cases where serum creatinine levels rise and drug-related nephrotoxicity cannot be ruled out, there is still a need to clarify how to adjust the dosage of PARPi or consider alternative types of PARP inhibitors.

## Data Availability

The original contributions presented in this study are included in this article/supplementary material, further inquiries can be directed to the corresponding authors.
